# Association of State Medicaid Expansion With Rate of Uninsured Hospitalizations for Major Cardiovascular Events, 2009-2014

**DOI:** 10.1001/jamanetworkopen.2018.1296

**Published:** 2018-08-24

**Authors:** Ehimare Akhabue, Lindsay R. Pool, Clyde W. Yancy, Philip Greenland, Donald Lloyd-Jones

**Affiliations:** 1 Department of Preventive Medicine, Northwestern University Feinberg School of Medicine, Chicago, Illinois; 2 Division of Cardiology, Department of Medicine, Northwestern University Feinberg School of Medicine, Chicago, Illinois

## Abstract

**Importance:**

Cardiovascular disease is the leading primary diagnosis among all hospital discharges, and insurance status is associated with patient outcomes. The association of state-level policy decisions regarding the Affordable Care Act (ACA) Medicaid expansion with rates of uninsured hospitalizations for major cardiovascular events and in-hospital mortality has not been investigated to date.

**Objective:**

To investigate whether the rates of uninsured hospitalizations for major cardiovascular events and in-hospital mortality varied by state-level policy on ACA Medicaid expansion.

**Design, Setting, and Participants:**

For this cohort study, difference-in-differences analysis of data from the Healthcare Cost and Utilization Project State Inpatient Databases of 30 US states on 524 848 non-Medicare hospitalizations in 2014 and a mean of 516 811 non-Medicare hospitalizations per year from 2009 to 2013 was performed for major cardiovascular events (defined as a composite of acute myocardial infarction, stroke, and heart failure) from January 1, 2009, through December 31, 2014. Analyses were completed September 1, 2017.

**Exposure:**

State Medicaid expansion as of January 1, 2014.

**Main Outcomes and Measures:**

Comparison of mean payer mix proportions (uninsured, Medicaid, and privately insured) and in-hospital mortality between expansion and nonexpansion states for the years preceding the ACA Medicaid expansion (2009-2013) and the year after the ACA Medicaid expansion (2014).

**Results:**

Of the 801 819 hospitalizations in the 17 expansion states in 2014, 428 503 (53.4%) patients were men, 514 036 (64.1%) were white, and 365 797 (45.6%) were aged 65 to 84 years. Of 719 459 hospitalizations in the 13 nonexpansion states in 2014, 383 311 (53.3%) patients were men, 492 136 (68.4%) were white, and 335 781 (46.7%) were aged 65 to 84 years. There were 281 184 non-Medicare hospitalizations for major cardiovascular events in the 17 expansion states and 243 664 non-Medicare hospitalizations in the 13 nonexpansion states in 2014. In multivariable regression analyses, the expansion states had a significant 5.8–percentage point decrease in the proportion of uninsured hospitalizations after Medicaid expansion relative to the nonexpansion states (adjusted difference-in-differences estimate, −0.058; 95% CI, −0.075 to −0.042; *P* < .001). The expansion states also had a significant 8.4–percentage point increase in the Medicaid share after Medicaid expansion relative to the nonexpansion states (adjusted difference-in-differences estimate, 0.084; 95% CI, 0.065 to 0.102; *P* < .001). In-hospital mortality did not change significantly after Medicaid expansion in either the expansion states (before ACA, 3.8% vs after ACA, 3.7%) or the nonexpansion states (4.0% vs 4.0%).

**Conclusions and Relevance:**

States that expanded Medicaid during the ACA implementation had a significantly greater reduction in the proportion of uninsured hospitalizations for major cardiovascular events compared with the nonexpansion states. This study suggests that expansion status was not associated with in-hospital mortality rates in the first year after ACA implementation.

## Key Points

**Question:**

Did uninsured hospitalizations for major cardiovascular events and in-hospital mortality vary by state-level policy decisions on the implementation of the Affordable Care Act Medicaid expansion?

**Findings:**

In this cohort study, difference-in-differences analysis of more than 3 million non-Medicare hospitalizations from the inpatient databases of 30 states found that expansion states had a significant reduction in the proportion of uninsured hospitalizations for major cardiovascular events within 1 year of Affordable Care Act Medicaid expansion compared with nonexpansion states. There were no changes in in-hospital mortality rates in expansion or nonexpansion states.

**Meaning:**

Further research is necessary to determine how state-level policy regarding Medicaid expansion could differentially affect cardiovascular outcomes.

## Teaser

This cohort study uses data from the HCUP Project State Inpatient Databases to compare the mean payer mix proportions and in-hospital mortality rates of 17 expansion states vs 13 nonexpansion states for the 4 years preceding and the year after the Affordable Care Act Medicaid expansion.

## Introduction

Cardiovascular disease (CVD) is the leading primary hospital discharge diagnosis and the most common cause of death in the United States.^1,2^ Coronary heart disease, stroke, and congestive heart failure represent the most common causes of CVD-related hospitalization.^3^ Individuals with markers of low socioeconomic status bear a disproportionate burden of CVD, including coronary heart disease and stroke, and are more likely to be uninsured.^2,4^ Access to adequate care is essential in major cardiovascular (CV) events. Insurance status is associated with clinical outcomes.^5,6,7,8,9^

It has been reported that since the first open enrollment period in October 2013, the Affordable Care Act (ACA) has resulted in millions of previously uninsured Americans acquiring health insurance coverage.^10,11,12^ The health insurance exchanges, subsidies, employer requirements for coverage, and Medicaid eligibility expansion have each contributed to these changes; however, not all states have opted to expand Medicaid.^12^ Previous data suggest that the ACA has improved access to health care clinicians and helped to reduce financial barriers to care, with the most significant gains occurring in expansion states—especially among low-income adults.^13,14,15,16,17^ Previous studies have examined ACA-related shifts in insurance payer mix for all hospitalizations in general and reported a significant reduction in the proportion of uninsured hospital discharges in Medicaid expansion states vs nonexpansion states.^18,19^ However, to our knowledge, the association between state-level policy regarding Medicaid expansion and uninsured hospitalizations for major CV events has not been investigated.

Whereas it might be expected that there would be fewer uninsured CVD-related hospitalizations after ACA implementation, it is important to document whether this was observed. It is also important to quantify the magnitude of change given the prominent role of CVD in overall public health and as the leading source of US medical expenditures.^2^ This is especially the case given data suggesting that uniformity of changes in payer proportions across different types of hospitalizations should not be presumed.^20^

Given the potential for CVD-related morbidity along with data suggesting mortality may have recently increased,^21^ understanding the association of particular components of the ACA with uninsured hospitalizations for major CV events has potentially significant health policy implications—especially in the non-Medicare population, in whom insurance coverage is less uniform and often closely linked to socioeconomic status. Furthermore, there is currently a dearth of data on the possible associations of expanded insurance coverage with CV outcomes. We sought to investigate whether the rates of uninsured hospitalizations for major CV events varied by state-level policy on Medicaid expansion. We further examined rates of in-hospital mortality over time as a potential indicator of changes in severity of presentation and level of care provided that may have been associated with Medicaid expansion.

## Methods

For this study, we used publicly available data for January 1, 2009, through December 31, 2014, from the Agency for Healthcare Research and Quality Healthcare Cost and Utilization Project State Inpatient Databases.^22^ Each participating Healthcare Cost and Utilization Project State Inpatient Database^22^ provided a census of actual inpatient hospitalizations specific to each state; this was distinct from the National Inpatient Sample,^23^ which provided estimates of hospitalizations and was not intended for state-level analyses. We collected data through the Healthcare Cost and Utilization Project Network^24^ online query engine, which provided aggregated patient information at the state level by year, including data on age, sex, race/ethnicity, payer (uninsured, Medicaid, Medicare, or privately insured) and residential classification (rural vs urban). Data were available through December 31, 2014, which was the first full year after implementation of ACA provisions including the individual insurance mandate and the Medicaid expansion. We chose 2009 as the beginning of our study period since this year preceded the signing of the ACA into law in 2010. This study was approved by the institutional review board at Northwestern University, Chicago, Illinois. Because we used publicly available deidentified data, the study was exempt from review by the Northwestern University institutional review board. This study followed the Strengthening the Reporting of Observational Studies in Epidemiology (STROBE) reporting guideline to the extent possible allowed by this type of analysis. Analyses were completed September 1, 2017, incorporating data available publicly through this period.

We identified hospitalizations for major CV events, defined as a composite of diagnoses that included acute myocardial infarction, stroke, and heart failure. These were based on prespecified *International Classification of Diseases, Ninth Revision* (*ICD-9*) diagnosis codes listed as the principal discharge diagnosis. We identified all hospitalizations in each state that had available data with the following *ICD-9* diagnosis codes listed as the principal diagnosis on discharge: acute myocardial infarction (410.x0 and 410.x1), stroke (430, 431, 433.x1, 434.x1, and 435-436), and heart failure (402.x1, 404.x1, 404.x3, and 428.x). These *ICD-9* diagnosis codes have shown good positive predictive value for these conditions when listed as the principal discharge diagnosis.^25,26,27,28^

Data were publicly available for 35 states. We excluded states that did not have hospitalization data available in 2014 (Massachusetts and New Hampshire) or that did not have relevant demographic data available for any year (Nebraska and Minnesota). We categorized states by their Medicaid expansion status in 2014, excluding Michigan where Medicaid expansion did not become effective until April 1, 2014 (rather than January 1, 2014). The state of Indiana did not expand Medicaid until after 2014 and was categorized as a nonexpansion state. Data from the 17 states (Arizona, Arkansas, California, Colorado, Hawaii, Illinois, Iowa, Kentucky, Maryland, Nevada, New Jersey, New Mexico, New York, Oregon, Rhode Island, Vermont, and Washington) that did expand (expansion states) and the 13 states (Florida, Indiana, Kansas, Maine, Missouri, North Carolina, Oklahoma, South Carolina, Tennessee, Texas, Utah, Wisconsin, and Wyoming) that did not expand (nonexpansion states) by 2014 were aggregated together as expansion and nonexpansion states. The final sample consisted of all hospitalizations for major CV events from 2009 through 2014 for 29 of these 30 states, and from 2011 through 2014 for 1 state (Indiana) that did not have 2009 and 2010 data available. We did not exclude Indiana since it had one of the larger populations and sufficient data were available for analysis of associations before ACA.

### Statistical Analysis

To assess the possible association of the ACA Medicaid expansion with uninsured hospitalizations for major CV events, we first calculated the proportion of the total hospitalizations for the expansion and nonexpansion states by insurance payer type. Because expansion of coverage was not expected to change in the Medicare population—which provided near-universal coverage for US adults older than 65 years even prior to the ACA—we limited our analyses to non-Medicare hospitalizations by subtracting Medicare-payer hospitalizations from the total number of hospitalizations. From this denominator of non-Medicare hospitalizations, we calculated the proportions of uninsured, Medicaid, and privately insured payer types.

Using a difference-in-differences approach, we constructed multivariable linear regression models to compare the mean payer mix from the years preceding Medicaid expansion (2009-2013) and the year after Medicaid expansion (2014) between expansion and nonexpansion states. Separate models were estimated for each payer status and adjusted for the time-varying state-level demographics (proportions of individuals who were female, <65 years, non-Hispanic white, and living in a rural location) of those hospitalized for major CV events. All models included a fixed effect for each state to account for state-level variation and were weighted by the total number of hospital discharges. Fixed-effects models also account for state-level correlation by calculating a separate, fixed intercept for each state.^29^ We also calculated the unadjusted change in hospitalization payer mix from 2013 to 2014 for each state and presented these data graphically. In sensitivity analyses (eTable in the Supplement), we repeated the difference-in-differences models to include the Medicare hospitalizations for major CV events since Medicaid expansion would not be expected to affect the proportion of Medicare hospitalizations. To assess for a possible association between Medicaid expansion and clinical outcomes, we also calculated the total number of in-hospital deaths among non-Medicare hospitalizations in the expansion and nonexpansion states for each study year. We then calculated the overall in-hospital mortality rate for each study year to investigate whether there was a difference over time by expansion status. Two-sided *P* values less than .05 were deemed to be statistically significant. All statistical analyses were performed using SAS, version 9.4 (SAS Institute Inc).

## Results

The demographic data for all major CV hospitalizations in expansion and nonexpansion states in the periods before ACA (2009-2013) and after ACA (2014) are presented in Table 1. Of the 801 819 hospitalizations in the expansion states in 2014, 428 503 (53.4%) patients were men, 514 036 (64.1%) were white, and 365 797 (45.6%) were aged 65 to 84 years. Of 719 459 hospitalizations in the nonexpansion states in 2014, 383 311 (53.3%) patients were men, 492 136 (68.4%) were white, and 335 781 (46.7%) were aged 65 to 84 years. In the 17 expansion states, the mean total number of non-Medicare hospitalizations during the period before ACA was 278 612, and the total number of non-Medicare hospitalizations in the first year after ACA (2014) was 281 184. In the 13 nonexpansion states, the mean total number of non-Medicare hospitalizations during the period before ACA was 238 199, and the total number of non-Medicare hospitalizations in the first year after ACA was 243 664. Overall, there were more hospitalizations in rural locations in the nonexpansion states than in the expansion states.

**Table 1.** All Hospitalizations for Major Cardiovascular Events by State Medicaid Expansion Status Before ACA (2009-2013) and After ACA (2014) Medicaid Expansion

Characteristic	Hospitalizations, No. (%)^a^			
	Expansion States^b^		Nonexpansion States^c^	
	Before ACA	After ACA	Before ACA	After ACA
All hospital discharges, No.	811 290^d^	801 819	704 325^d^	719 459
Age group, y				
18-44	38 231 (4.7)	37 734 (4.7)	35 682 (5.1)	36 362 (5.1)
45-64	230 286 (28.4)	235 352 (29.4)	211 681 (30.1)	219 158 (30.5)
65-84	372 671 (45.9)	365 797 (45.6)	328 552 (46.6)	335 781 (46.7)
≥85	168 902 (20.8)	161 714 (20.2)	127 220 (18.1)	127 023 (17.7)
≤17 or missing data	1200 (0.1)	1222 (0.2)	1190 (0.2)	1135 (0.2)
Sex				
Male	421 559 (52.0)	428 503 (53.4)	367 098 (52.1)	383 311 (53.3)
Female	389 716 (48.0)	373 300 (46.6)	337 181 (47.9)	336 110 (46.7)
Missing data	14 (<0.01)	16 (<0.01)	46 (0.01)	38 (0.01)
Insurance payer				
Medicare	532 678 (65.7)	520 635 (64.9)	466 126 (66.2)	475 795 (66.1)
Medicaid	71 268 (8.8)	98 819 (12.3)	43 398 (6.2)	45 278 (6.3)
Private insurance	151 064 (18.6)	146 128 (18.2)	128 338 (18.2)	130 814 (18.2)
Uninsured	36 422 (4.5)	21 564 (2.7)	49 212 (7.0)	48 787 (6.8)
Other^e^	19 030 (2.3)	13 848 (1.7)	16 189 (2.3)	17 706 (2.5)
Missing data	828 (0.1)	825 (0.1)	1062 (0.2)	1079 (0.2)
Race/ethnicity				
White	530 764 (65.4)	514 036 (64.1)	489 555 (69.5)	492 136 (68.4)
Black	114 059 (14.1)	112 041 (14.0)	115 894 (16.5)	122 378 (17.0)
Hispanic	83 601 (10.3)	88 511 (11.0)	63 442 (9.0)	66 609 (9.3)
Asian/Pacific Islander	36 526 (4.5)	39 475 (4.9)	5091 (0.7)	5600 (0.8)
Native American	3927 (0.5)	3880 (0.5)	4414 (0.6)	4730 (0.7)
Other^f^	28 416 (3.5)	30 530 (3.8)	17 565 (2.5)	17 022 (2.4)
Missing data	13 996 (1.7)	13 346 (1.7)	8365 (1.2)	10 984 (1.5)
Location (residence)				
Rural	109 825 (13.5)	96 827 (12.1)	167 640 (23.8)	150 179 (20.9)
Urban/suburban	697 696 (86.0)	701 331 (87.5)	534 280 (75.9)	567 070 (78.8)
Missing data	3768 (0.5)	3361 (0.5)	2405 (0.3)	2210 (0.3)

Abbreviation: ACA, Affordable Care Act.

^a^ Column percentages may not add up to 100% because of rounding.

^b^ Based on data from 17 states: Arizona, Arkansas, California, Colorado, Hawaii, Illinois, Iowa, Kentucky, Maryland, Nevada, New Jersey, New Mexico, New York, Oregon, Rhode Island, Vermont, and Washington.

^c^ Based on data from 13 states: Florida, Indiana, Kansas, Maine, Missouri, North Carolina, Oklahoma, South Carolina, Tennessee, Texas, Utah, Wisconsin, and Wyoming.

^d^ Mean during 2009-2013 period. The mean for nonexpansion states is weighted to account for no available data from the state of Indiana for 2009 and 2010.

^e^ Payers not categorized as Medicare, Medicaid, or commercial programs as reported by each state.

^f^ Race/ethnicity not categorized as white, black, Hispanic, Asian/Pacific Islander, or Native American as reported by each state.

**Figure 1.**
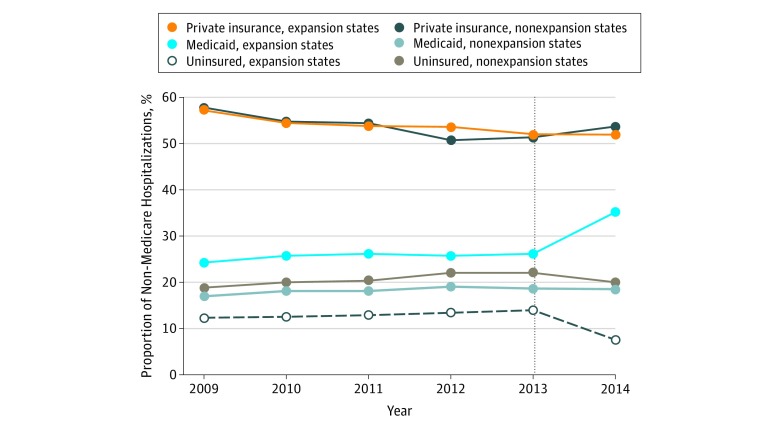
Payer Proportion of Non-Medicare Hospitalizations for Major Cardiovascular Events by Expansion Status, 2009-2014 The dotted vertical line after year 2013 represents implementation of the Affordable Care Act that included Medicaid expansion. Data presented are for the completion of each year. Data for nonexpansion states in 2009 and 2010 do not include the state of Indiana. After Affordable Care Act implementation, there was a significant change in the yearly uninsured and Medicaid payer proportions in expansion states.

Payer mix for each year from 2009 to 2014 for non-Medicare hospitalizations in the expansion and nonexpansion states is presented in Figure 1. After ACA implementation, there was a significant change in the yearly uninsured and Medicaid proportions of non-Medicare hospitalizations in expansion states. Throughout the study period, the proportion of uninsured hospitalizations for each year was consistently higher and the proportion of Medicaid hospitalizations consistently lower in the nonexpansion states compared with the expansion states, with an increase in these differences after ACA implementation (Figure 1). Payer mix composition differed by type of major CV event (myocardial infarction, stroke, or heart failure), but similar shifts in payer mix for uninsured and Medicaid proportions of non-Medicare hospitalizations for each type of major CV event were seen after ACA implementation (eFigure in the Supplement).

In crude unadjusted analyses, after ACA implementation, the uninsured proportion of non-Medicare hospitalizations decreased by 5.4 percentage points after ACA vs before ACA (mean before ACA: 36 422 of 278 612 [13.1%] vs after ACA: 21 564 of 281 184 [7.7%]), whereas the Medicaid proportion increased by 9.5 percentage points (before ACA: 71 268 of 278 612 [25.6%] vs after ACA: 98 819 of 281 184 [35.1%]) in the expansion states. In the nonexpansion states, similar differences were not seen. The uninsured proportion of non-Medicare hospitalizations before ACA was 49 212 of 238 199 (20.7%) and after ACA was 48 787 of 243 664 (20.0%), whereas the Medicaid proportion before ACA was 43 998 of 238 199 (18.2%) and after ACA was 45 278 of 243 664 (18.6%).

In regression analyses, the uninsured proportion of non-Medicare hospitalizations decreased significantly by 5.0 percentage points after ACA vs before ACA (difference estimate, −0.050; 95% CI, −0.062 to −0.038; *P* < .001) (Table 2), whereas the Medicaid proportion increased significantly by 10.2 percentage points (0.102; 95% CI, 0.088 to 0.116; *P* < .001). In the nonexpansion states, the uninsured proportion did not change significantly (difference estimate, 0.003 [or 0.3 percentage points]; 95% CI, −0.011 to 0.017), nor did the Medicaid proportion (0.009 [or 0.9 percentage points]; 95% CI, −0.003 to 0.020), consistent with the findings in the raw analyses.

**Table 2.** Change in Payer Proportion for Non-Medicare Hospitalizations for Major Cardiovascular Events by State Medicaid Expansion Status Before ACA (2009-2013) and After ACA (2014) Medicaid Expansion

Type of Hospital Discharge^a^	Payer Proportion				
	Medicaid Expansion		Difference (95% CI)	Difference-in-Differences (95% CI)^b^	
	Before ACA	After ACA		Unadjusted	Adjusted
Uninsured					
Expansion	0.126	0.077	−0.050 (−0.062 to −0.038)^c^	−0.053 (−0.071 to −0.035)^c^	−0.058 (−0.075 to −0.042)^c^
Nonexpansion	0.202	0.200	0.003 (−0.011 to 0.017)		
Medicaid					
Expansion	0.251	0.351	0.102 (0.088 to 0.116)^c^	0.093 (0.075 to 0.112)^c^	0.084 (0.065 to 0.102)^c^
Nonexpansion	0.177	0.186	0.009 (−0.003 to 0.020)		
Private insurance					
Expansion	0.553	0.520	−0.033 (−0.052 to −0.015)^c^	−0.019 (−0.047 to 0.010)	−0.007 (−0.029 to 0.016)
Nonexpansion	0.550	0.537	−0.015 (−0.037 to 0.008)		

Abbreviation: ACA, Affordable Care Act.

^a^ Separate regression models were generated for each payer status. All models included a fixed effect for state to account for state-level variation and were weighted by total number of discharges. Multivariable models were adjusted for time-varying state-level demographics of all discharges for major cardiovascular events, including proportions of individuals who were female, younger than 65 years, non-Hispanic white, and living in a rural location.

^b^ Proportion estimates are directly convertible to percentage points by multiplying by 100.

^c^ *P* < .001.

In the multivariable difference-in-differences regression analyses, the expansion states had a significant 5.8–percentage point decrease in the uninsured proportion of non-Medicare hospitalizations after expansion relative to the nonexpansion states (adjusted difference-in-differences estimate, −0.058; 95% CI, −0.075 to −0.042; *P* < .001) (Table 2) along with a significant 8.4–percentage point increase in the proportion of Medicaid hospitalizations after expansion relative to the nonexpansion states (0.084; 95% CI, 0.065 to 0.102; *P* < .001). There was no significant change in the private share of hospitalizations in the expansion states relative to the nonexpansion states (−0.007 [or −0.7 percentage points]; 95% CI, −0.029 to 0.016; *P* = .54)

In sensitivity analyses with Medicare hospitalizations included in the population, the expansion states still had a significant decrease in proportion of uninsured hospitalizations after expansion compared with the nonexpansion states (adjusted difference-in-differences estimate, −0.019 [or −1.9 percentage points]; 95% CI, −0.024 to −0.014; *P* < .001) along with a significant increase in proportion of Medicaid hospitalizations after expansion compared with the nonexpansion states (0.031 [or 3.1 percentage points]; 95% CI, 0.025 to 0.038; *P* < .001). As would be expected, there were no significant changes in the proportion of Medicare hospitalizations in the expansion states or the nonexpansion states after Medicaid expansion (eTable in the Supplement).

**Figure 2.**
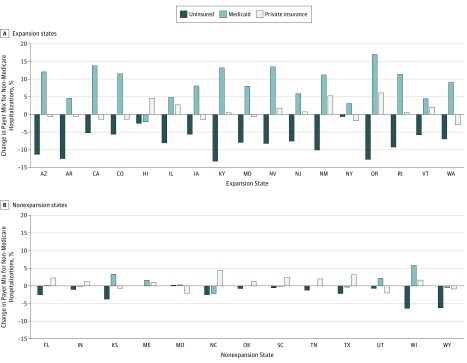
Relative Changes in Proportion of Uninsured, Medicaid, and Private Insurance Non-Medicare Hospitalizations for Major Cardiovascular Events by State, 2013-2014 A, Uninsured hospitalizations vs Medicaid and private insurance in expansion states. B, Uninsured hospitalizations vs Medicaid and private insurance in nonexpansion states. Concurrent with the decrease in uninsured hospitalizations, the proportion of Medicaid hospitalizations increased in most expansion states whereas this pattern was not seen consistently in the nonexpansion states. AR indicates Arkansas; AZ, Arizona; CA, California; CO, Colorado; FL, Florida; HI, Hawaii; IA, Iowa; IL, Illinois; IN, Indiana; KS, Kansas; KY, Kentucky; MD, Maryland; ME, Maine; MO, Missouri; NC, North Carolina; NJ, New Jersey; NM, New Mexico; NV, Nevada; NY, New York; OK, Oklahoma; OR, Oregon; RI, Rhode Island; SC, South Carolina; TN, Tennessee; TX, Texas; UT, Utah; VT, Vermont; WA, Washington; WI, Wisconsin; and WY, Wyoming.

Figure 2 presents unadjusted individual state-level changes in payer mix between 2013 and 2014. The proportion of uninsured hospitalizations decreased in most of the individual states but these changes tended to be greater in the expansion states, concurrent with increases in Medicaid share, whereas this pattern was not seen consistently in the nonexpansion states.

The percentages of in-hospital deaths per year among non-Medicare hospitalizations in the expansion and nonexpansion states are presented in Table 3. In regression analyses, there was no significant change in in-hospital mortality after ACA implementation in either the expansion states (3.7% in 2014 vs 3.8% over the 2009-2013 period; *P* = .30) or the nonexpansion states (4.0% in 2014 vs 4.0% over the 2009-2013 period; *P* = .39), nor was there a significant difference in in-hospital deaths in the expansion states relative to the nonexpansion states when comparing the periods before and after ACA (adjusted difference-in-differences estimate, −0.001 [or −0.1 percentage points]; 95% CI, −0.002 to 0.001; *P* = .42).

**Table 3.** In-Hospital Mortality for Non-Medicare Hospitalizations of Major Cardiovascular Events by State Medicaid Expansion Status, 2009-2014

Status	2009	2010	2011	2012	2013	2014
Expansion states^a^						
Non-Medicare hospital discharges, No.	283 796	285 897	275 066	274 498	273 802	281 184
In-hospital death, No. (%)	10 813 (3.8)	10 729 (3.8)	10 322 (3.8)	10 255 (3.7)	10 294 (3.8)	10 441 (3.7)
Nonexpansion states^b^						
Non-Medicare hospital discharges, No.	227 090^c^	227 848^c^	242 633	226 887	233 190	243 664
In-hospital death, No. (%)	9175 (4.0)^c^	9258 (4.1)^c^	9589 (4.0)	8670 (3.8)	9059 (3.9)	9701 (4.0)

^a^ Based on data from 17 states: Arizona, Arkansas, California, Colorado, Hawaii, Illinois, Iowa, Kentucky, Maryland, Nevada, New Jersey, New Mexico, New York, Oregon, Rhode Island, Vermont, and Washington.

^b^ Based on data from 13 states: Florida, Indiana, Kansas, Maine, Missouri, North Carolina, Oklahoma, South Carolina, Tennessee, Texas, Utah, Wisconsin, and Wyoming.

^c^ Does not include data from the state of Indiana, which were not available for 2009 and 2010.

## Discussion

### Principal Findings

In this quasi-experimental difference-in-differences cohort study, we examined the potential associations between the ACA implementation and the rates of uninsured hospitalizations for 3 of the top causes of CVD-related morbidity and mortality in the United States. We found that, after full implementation of the major provisions of the ACA in the beginning of 2014, there was a substantially greater decline in the proportion of uninsured hospitalizations for the major CV events under study in states that expanded Medicaid vs those states that did not. The distinct change in the proportion of hospitalizations that were uninsured and covered by Medicaid in the expansion states suggested that significant decreases in uninsured hospitalizations for major CV events were associated with Medicaid expansion. That these findings were already present within 1 year of ACA implementation suggested that the changes in payer mix were immediate. To our knowledge, our study is the first to document these observations for major CV events and to quantify the magnitude of these potential associations. This is important given that one could not presume that these changes in payer proportions would have been uniformly present across different types of hospitalizations.^20^ Notably, these changes occurred without an immediate influence on in-hospital mortality.

### Present Study in Context

Our findings on payer mix for CV hospitalizations were consistent with previous analyses examining the potential association of Medicaid expansion on coverage status for all non-Medicare hospitalizations.^18,19,30^ Our study extended previous analyses in 3 significant ways. First, we characterized these payer results by specifically focusing on hospitalizations for CVD, the leading cause of death and hospitalization in the United States. We investigated which aspects of the ACA may have had the most significant immediate effect on coverage for uninsured patients who would be hospitalized for major CV events. It has been reported that millions of individuals in the United States have acquired private health insurance coverage since the full implementation of the ACA.^10,11^ Nevertheless, our analyses did not show a substantial difference between the expansion and nonexpansion states in the private insurance share of CV discharges after expansion. Thus, our data suggested that, of the uninsured individuals who would be hospitalized for a major CV event and who acquired insurance coverage at that time, most qualified as low-income status and received access to Medicaid in the first year after full ACA implementation. Second, our study examined this focused question of whether there were associations between changes in payer mix for CVD hospitalizations and Medicaid expansion with hospitalization data from a large number of states, which together represented nearly three-quarters of the US population.^31^ Finally, we examined whether state expansion status was associated with in-hospital mortality for these major CV events, an important consideration when evaluating the potential association of insurance status with outcomes.

Previous data have shown that significant variations in CV health between states were contributed to not only by individual factors but also state-level factors, including policy.^32^ Previous data suggested that the ACA had led to improvements among low-income non-Medicare patients, such as having a primary care clinician, accessing routine medical care, and not forgoing medications because of cost—with Medicaid expansion being associated with the most substantial improvements.^14,15,16^ While previous studies have shown that insurance status can be tied to hospital care and outcomes after major CV events, there was an overall dearth of data directly comparing the CV outcomes of hospitalized patients with Medicaid coverage with those who were uninsured.^5,6,7,9^ We observed that in-hospital mortality did not change significantly after Medicaid expansion in our study. Greater decreases in uninsured hospitalizations did not translate to decreases in hospital death rates—which may not have been unexpected after only 1 year given that insurance coverage may take time to influence overall health status. However, in-hospital mortality also did not increase significantly despite the fact that ACA expansion provided coverage for more low-income persons and that lower socioeconomic status was associated with a higher prevalence of CVD and worse outcomes.^2^ Adequately exploring any potential associations of expanded coverage will require further research using more long-term data beyond what we had available for this study.

A major consideration of our findings is the potential implications of cost at the macroeconomic and microeconomic levels. Cardiovascular disease is the leading source of medical expenditures in the United States, with direct medical costs projected to approach nearly $1 trillion by 2030, of which more than half is currently the result of inpatient hospitalizations.^2^ By nature of near-universal Medicare coverage for people 65 years or older, it is nonelderly patients with CVD who are more likely to be uninsured. That Medicaid expansion was associated with a significant reduction in the proportion of uninsured hospitalizations may have had important out-of-pocket cost implications for low-income patients who would have been previously uninsured but now had access to Medicaid.

There was evidence that hospitals may have benefited from Medicaid expansion through decreases in uncompensated care costs.^33,34^ In contrast, the ACA also had provisions to decrease the disproportionate share hospital allotments, which supplemented the income of hospitals that take care of underinsured patients.^35^ In addition, any decrease in cost burden for individuals and hospitals because of the Medicaid expansion might be expected to shift to the states and the federal government. Thus, discussions of economic impact and costs must take into account all of these factors in addition to effects on hospital, state, and federal expenditures. Adequate time is unlikely to have passed to draw conclusions on the association of the ACA with costs related to CVD—the unclear direction of federal policy on health insurance coverage adds complexity to what is already a complicated assessment. Given the prominent role of CVD in overall public health, and as the leading source of medical expenditures, it will be important that future study takes into account the prominent role of CVD in addressing these questions.

### Limitations

Strengths of our study included the use of a large representative sample of the top 3 causes of CVD-related hospitalizations in the United States. Our analyses were based on data from the Healthcare Cost and Utilization Project State Inpatient Databases,^22^ each of which provided a census of all inpatient hospitalizations by state. We note that the change in the sampling design for the National Inpatient Sample^23^ after 2011 did not apply to our analyses. Nevertheless, our findings should be viewed with other limitations in mind. We used *ICD-9* diagnosis codes, which identified CVD as the principal diagnosis, to create our composite outcome. Despite good positive predictive value for identifying acute myocardial infarction, heart failure, and stroke, use of *ICD-9* diagnosis codes for these, as with other diagnoses, can have suboptimal sensitivity.^26,28,36^ It is possible that we underestimated the total number of hospitalizations for these events. In addition, our data did not represent individuals but rather encounters, and rehospitalizations were not distinguished from primary hospitalizations. Data were only available for 1 year after ACA implementation; thus, we could not determine whether the associations noted in this study would have continued to the present. We also could not reliably analyze the changes in total number of hospitalizations for major CV events after ACA implementation—another important consideration in the assessment of the ACA influence on CVD, which was beyond the scope of this study. We used data from a large number of states that together represented nearly three-quarters of the US population; nevertheless, we could not rule out the possibility that if data from additional states were available, it might have significantly changed our results. We adjusted for time-varying, state-level demographics and accounted for variation at the state level using a fixed-effects modeling strategy. Nevertheless, there may be some residual confounding based on time-varying, state-level health or economic trends that we were unable to adjust for because we did not have data specific to the hospitalized population.

## Conclusions

States that chose to expand Medicaid as part of the ACA implementation had a significantly greater reduction in the proportion of uninsured hospitalizations for major CV events compared with the nonexpansion states, but expansion status was not associated with in-hospital mortality in the first postexpansion year. Further research is necessary to determine how state-level policy decisions regarding ACA implementation could differentially influence short-term and long-term CV outcomes before, during, and after hospitalization.

## Notes

Supplement. **eTable.** Change in Payer Proportion for All Hospitalizations (Including Medicare) for Major Cardiovascular Events by State Medicaid Expansion Status, Pre-ACA (2009-2013) and Post-ACA (2014) Medicaid Expansion **eFigure.** Trends Over Time in Payer Proportion for Non-Medicare Hospitalizations for Stroke, Heart Failure (HF), and Myocardial Infarction (MI) by Expansion Status, 2009-2014
