# Supplementary material for: Association of State Medicaid Expansion With Rate of Uninsured Hospitalizations for Major Cardiovascular Events, 2009-2014
Source: JAMA Netw Open. 2018 Aug 24;1(4):e181296. doi: 10.1001/jamanetworkopen.2018.1296 (PMC6324285; doi:10.1001/jamanetworkopen.2018.1296)
Supplement: Supplement. — eTable. Change in Payer Proportion for All Hospitalizations (Including Medicare) for Major Cardiovascular Events by State Medicaid Expansion Status, Pre-ACA (2009-2013) and Post-ACA (2014) Medicaid Expansion eFigure. Trends Over Time in Payer Proportion for Non-Medicare Hospitalizations for Stroke, Heart Failure (HF), and Myocardial Infarction (MI) by Expansion Status, 2009-2014 [file jamanetwopen-1-e181296-s001.pdf]

# Supplementary Online Content

Akhabue E, Pool LR, Yancy CW, Greenland P, Lloyd-Jones D. Association of state Medicaid expansion with rate of uninsured hospitalizations for major cardiovascular events, 2009-2014. *JAMA Netw Open*. 2018;1(4):e181296. doi:10.1001/jamanetworkopen.2018.1296

**eTable.** Change in Payer Proportion for All Hospitalizations (Including Medicare) for Major Cardiovascular Events by State Medicaid Expansion Status, Pre-ACA (2009-2013) and Post-ACA (2014) Medicaid Expansion

**eFigure.** Trends Over Time in Payer Proportion for Non-Medicare Hospitalizations for Stroke, Heart Failure (HF), and Myocardial Infarction (MI) by Expansion Status, 2009-2014

This supplementary material has been provided by the authors to give readers additional information about their work.

© 2018 Akhabue E et al. *JAMA Network Open*.

**eTable.** Change in Payer Proportion for All Hospitalizations (Including Medicare) for Major Cardiovascular Events by State Medicaid Expansion Status, Pre-ACA (2009-2013) and Post-ACA (2014) Medicaid Expansion

| | Before Expansion | After expansion | Difference [95% CI] | Unadjusted DD [95% CI] | Adjusted DD [95% CI] |
|---|---|---|---|---|---|
| **Uninsured discharges** | | | | | |
| **Expansion** | 0.043 | 0.027 | -0.017 [-0.020, -0.013][a] | | |
| **Non-expansion** | 0.068 | 0.068 | 0.0001 [-0.004, 0.004] | | |
| **DD** | | | | -0.017 [-0.022, -0.012][a] | -0.019 [-0.024, -0.014][a] |
| **Medicaid discharges** | | | | | |
| **Expansion** | 0.086 | 0.123 | 0.038 [0.032, 0.043][a] | | |
| **Non-expansion** | 0.060 | 0.063 | 0.003 [-0.00002, 0.006] | | |
| **DD** | | | | 0.034 [0.028, 0.041][a] | 0.031 [0.025, 0.038][a] |
| **Private discharges** | | | | | |
| **Expansion** | 0.190 | 0.182 | -0.009 [-0.019, 0.001] | | |
| **Non-expansion** | 0.186 | 0.182 | -0.005 [-0.018, 0.009] | | |
| **DD** | | | | -0.004 [-0.020, 0.012] | 0.002 [-0.012, 0.015] |
| **Medicare Discharges** | | | | | |
| **Expansion** | 0.656 | 0.649 | -0.006 [-0.014, 0.003] | | |
| **Non-expansion** | 0.661 | 0.661 | 0.0004 [-0.012, 0.013] | | |
| **DD** | | | | -0.006 [-0.021, 0.008] | -0.008 [-0.021, 0.005] |

Separate regression models were generated for each payer status. All models included a fixed effect for state to account for state-level variation and were weighted by total number of discharges. Multivariable models adjusted for time-varying state-level demographics of all discharges for major cardiovascular events, including percent female, percent non-Hispanic white, percent living in rural areas, and percent under age 65.

Abbreviations: ACA, Affordable Care Act; CI, Confidence Interval; DD, Difference-in-Differences

[a]$p < 0.001$

Proportion estimates are directly convertible to percentage points by multiplying by 100

© 2018 Akhabue E et al. *JAMA Network Open*.

**eFigure.** Trends Over Time in Payer Proportion for Non-Medicare Hospitalizations for Stroke, Heart Failure (HF), and Myocardial Infarction (MI) by Expansion Status, 2009-2014

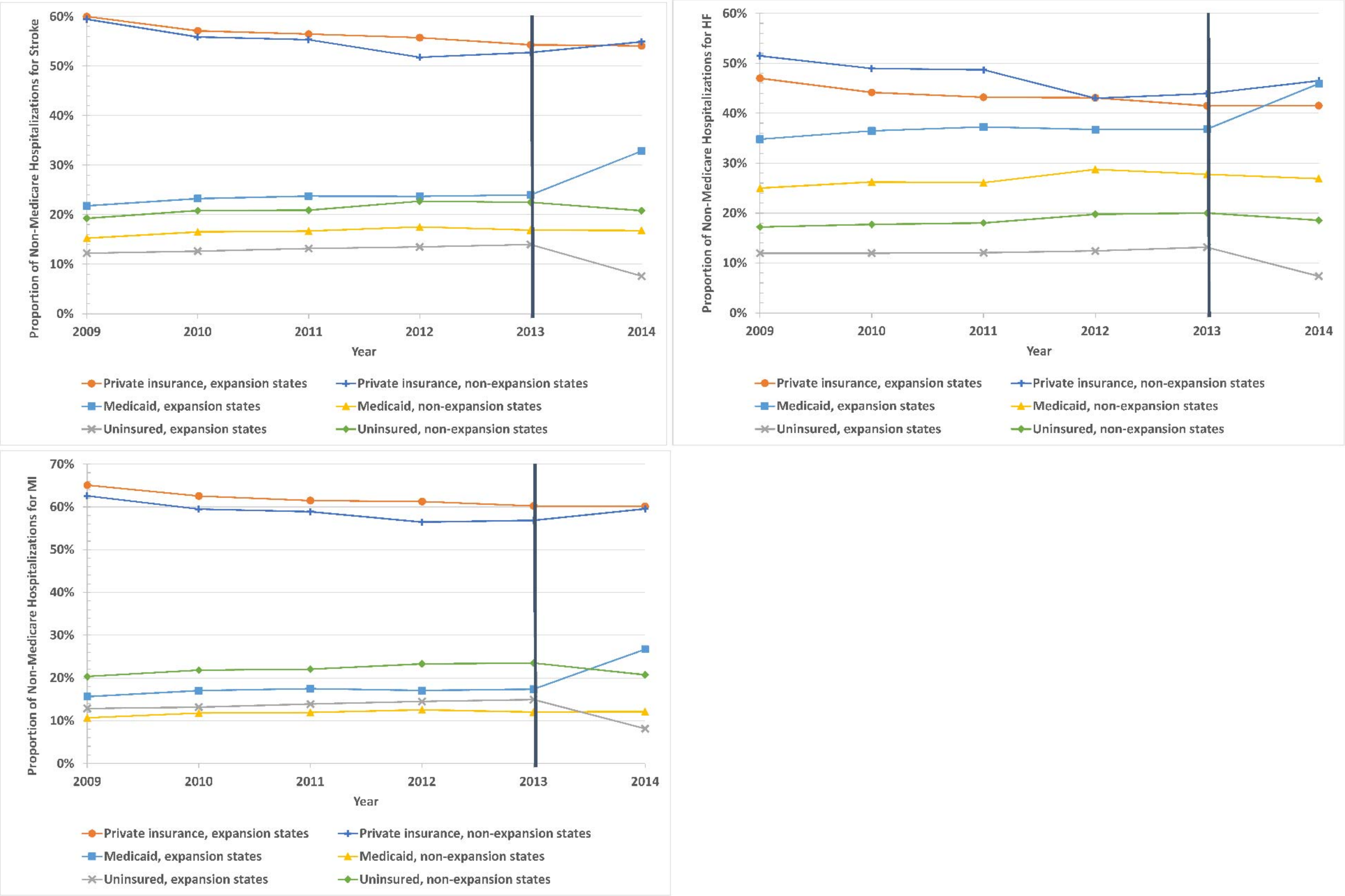


**The dark line represents implementation of the Affordable Care Act which included Medicaid expansion. Payer mix composition differed by individual type of cardiovascular event but similar shifts in trend for uninsured and Medicaid proportions of non-Medicare hospitalizations for each type of cardiovascular event were seen after ACA implementation.**

© 2018 Akhabue E et al. *JAMA Network Open*.
